# Bis-SNP: Combined DNA methylation and SNP calling for Bisulfite-seq data

**DOI:** 10.1186/gb-2012-13-7-r61

**Published:** 2012-07-11

**Authors:** Yaping Liu, Kimberly D Siegmund, Peter W Laird, Benjamin P Berman

**Affiliations:** 1 USC Epigenome Center, University of Southern California, 1450 Biggy Street, Los Angeles, CA 90089, USA; 2 Genetics, Molecular and Cellular Biology Program, University of Southern California, 1975 Zonal Avenue KAM-B16, Los Angeles, CA 90089, USA; 3 Department of Preventive Medicine, Keck School of Medicine, University of Southern California, 1441 Eastlake Avenue, Los Angeles, CA 90089, USA

## Abstract

Bisulfite treatment of DNA followed by high-throughput sequencing (Bisulfite-seq) is an important method for studying DNA methylation and epigenetic gene regulation, yet current software tools do not adequately address single nucleotide polymorphisms (SNPs). Identifying SNPs is important for accurate quantification of methylation levels and for identification of allele-specific epigenetic events such as imprinting. We have developed a model-based bisulfite SNP caller, Bis-SNP, that results in substantially better SNP calls than existing methods, thereby improving methylation estimates. At an average 30× genomic coverage, Bis-SNP correctly identified 96% of SNPs using the default high-stringency settings. The open-source package is available at http://epigenome.usc.edu/publicationdata/bissnp2011.

## Background

Cytosine methylation of DNA plays an important role in mammalian gene regulation, chromatin structure and imprinting during normal development and the development of pathological conditions such as cancer. With the dramatic increase in throughput made possible by next-generation DNA sequencing technologies, sodium bisulfite conversion followed by massively parallel sequencing (Bisulfite-seq) has become an increasingly popular method for investigating epigenetic profiles in the human genome (reviewed in [1]). Several sequencing strategies have been applied that vary in terms of cost and the regions of the genome covered. Reduced Representation Bisulfite-Seq (RRBS [2]) uses restriction fragment size selection to select a portion of the genome enriched for CpG Islands and gene regulatory sequences. Bisulfite Padlock Probes (BSPP [3]) or solution-based hybridization capture (Agilent, Inc., Santa Clara, CA, USA) can be designed for customizable selection of hundreds of thousands of regions throughout the genome. Whole-Genome Bisulfite-Seq (WGBS [4]) is the most comprehensive technique, covering more than 90% of cytosines in the human genome. Bisulfite-seq is well-suited to the investigation of epigenetic changes from clinical tissue samples [5,6], and can be applied to very small quantities of DNA [7] including formalin-fixed samples [8]. WGBS and RRBS data have been used to profile a number of cell lines and human tissues by large sequencing consortia including the ENCODE project [9], the NIH Epigenomics Roadmap, and The Cancer Genome Atlas (TCGA), and these datasets are publicly available for download.

Bisulfite treatment of DNA converts unmethylated cytosines to uracils, which are replaced by thymines during amplification. This dramatic change to sequence composition necessitates specialized software for almost all sequence analysis tasks. Typically, the first step in processing high-throughput sequencing data is to map and align each read to the correct location in the reference genome (genome mapping), and a number of powerful tools have been developed to map bisulfite-converted reads (reviewed in [10]). The next step is to identify differences between the reference genome and the sample genome, including single-nucleotide polymorphisms (SNPs) and insertion/deletion events (indels). The identification of SNPs has been an active area of research and a number of powerful statistical tools have been developed for SNP calling of non-bisulfite sequencing data [11-13]. SNP calling of bisulfite sequencing data has significant complications. First, reads from the two genomic strands are not complementary, and this assumption of complementarity is made by all SNP calling algorithms. Second, true (evolutionary) C>T SNPs in the sample cannot be distinguished from C>T substitutions that are caused by bisulfite conversion, and can thus be misidentified as unmethylated Cs. Consequently, identification of such SNPs is important for accurate quantification of methylation levels, especially so given the fact that C>T is the most common substitution in the human population (65% of all SNPs in dbSNP) and these usually occur in the CpG context [14].

Accurate SNP calling at the positions immediately surrounding a cytosine is equally important. Those nucleotides lying one or two positions 3' of the cytosine are particularly critical, as they are subject to the specificity of particular methyltransferases. These methyltransferase-specific context positions can be organism or cell type specific. In mammals, CpG dinucleotides are often highly methylated in most cell types, while CpA dinucleotides have much lower methylation levels and are cell type restricted [4,15]. In plants, by contrast, CHG trinucleotides are often methylated [16,17]. Other sequences within a slightly wider genomic neighborhood can also have strong *cis *effects on methylation, perhaps due to the presence of key regulatory motifs [18]. Heterozygous SNPs in proximity to cytosines can be used to reveal widespread allele-specific methylation patterns [19] and important regulatory changes such as loss of imprinting [20-22].

Despite the great interest in Bisulfite-seq and the availability of a number of tools for genomic mapping, no adequate software exists for SNP calling [10]. In order to overcome the difficulty in identifying SNPs in bisulfite-treated sequences, some groups have relied on matched non-bisulfite sequencing data in the same sample [23-25]. Others have used non-bisulfite SNP microarrays [26,27], or used study designs relying on isogenic mouse strains with known parental genotypes [22,24].

**Figure 1:**
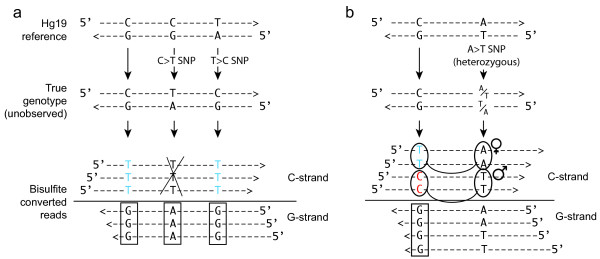
**Detecting single nucleotide polymorphisms from Bisulfite-seq data**. Hypothetical bisulfite-sequencing data is shown, with reference genome at top, genome of the individual sequenced (unobserved) in the middle, and bisulfite sequencing reads bottom. (**a**) shows three reference cytosine positions, with the first being a match to the reference genome and the second two being *homozygous *single nucleotide polymorphisms. The first case shows a true C:G genotype, and all reads on the same strand as the C (the 'C-strand') are read as T, indicating an unmethylated state (shown as blue). Because the Illumina Bisulfite-seq protocol is 'directional', reads on the opposite strand (the 'G-strand') are read as the true genotype, G ('genotype' reads on the G-strand are boxed in this figure). The second case illustrates a true C>T SNP, which can be distinguished by the A reads present on the G-strand. In this case, the reads on the C-strand are inferred to be from a true 'T' and should *not *be used for methylation calling (crossed out here). The third case shows a T>C SNP, which again can be identified based on G-strand reads. (**b**) A cytosine position with 50% unmethylated (T) and 50% methylated (C) reads can be associated with a heterozygous SNP on the same sequencing reads. In this case, the unmethylated reads are those on the 'A' allele chromosome (here shown as maternal) and the methylated reads are on the 'T' allele chromosome.

A key property of some bisulfite-related protocols is that G nucleotides on the strand opposing a C are not affected by conversion. This strand-specificity principle has been exploited in order to distinguish bisulfite conversion from C>T SNPs [28]. The Illumina-based protocol currently being used in most Bisulfite-seq studies has this important property, and thus it has been classified as a *directional *bisulfite-seq protocol [10]. *Non-directional *protocols (those that also result in G>A substitutions) have been used [17], but have not been widely adopted. Figure 1 illustrates the directional protocol, where approximately half the reads at a given cytosine position (those mapping to the 'C-strand') can be used for methylation quantification but cannot distinguish C>T SNPs. The other half (those mapping to the 'G-strand', boxed in Figure 1a) yield no methylation information but can be used to identify C>T SNPs. When these C>T SNPs are heterozygous, they can be used in the analysis of allele specific methylation (Additional File 1).

The inherent directionality of Illumina Bisulfite-seq has thus far been used only in a limited and *ad hoc *way. The Salk Institute group filtered out cytosines which did not have one or more unconverted Cs on the C-strand, but this approach can result in lost information about completely unmethylated cytosines (which play a crucial role in gene regulation) [4,29]. Our own group filtered out reference Cs if opposing reads contained As, but the number of such A reads required was somewhat arbitrary [6]. A third group removed all C/T reads on the C-strand, and called SNPs by requiring a minimum number of reads containing two different alleles [30]. Importantly, none of these so-called 'k-allele' approaches took advantage of base calling quality scores, which have been shown to be extremely important for distinguishing true SNPs from sequencing errors [31]. Others used various methods that did not attempt to identify C/T or other SNPs occurring at cytosines [3,20,21]. Such methods may be useful for analyzing allele-specific patterns in a limited way, but do not address the need to improve methylation quantification by identifying SNPs.

Here, we describe a probabilistic SNP caller, Bis-SNP, that is based on methods that have proven successful in non-bisulfite SNP calling [12,13]. Bis-SNP uses Bayesian inference to evaluate a model of strand-specific base calls and base call quality scores, along with prior information on population SNP frequencies, experiment-specific bisulfite conversion efficiency, and site-specific DNA methylation estimates. It also takes advantage of base call quality score recalibration, an addition that has greatly improved SNP calling in the non-bisulfite context [12]. Bis-SNP is open-source and based on the GATK framework [32], which takes advantage of the parallel Map-Reduce computation strategy and provide practical execution times. Bis-SNP accepts either single-end or paired-end mapped Bisulfite-seq data in the form of BAM files, and outputs SNP and methylation information using standard file formats. We show that Bis-SNP is a practical tool that can both (1) improve DNA methylation calling accuracy by detecting SNPs at cytosines and adjacent positions, and (2) identify heterozygous SNPs that can be used to investigate mono-allelic DNA methylation and polymorphisms in cis-regulatory sequences.

## Results and discussion

### Bis-SNP workflow

**Figure 2:**
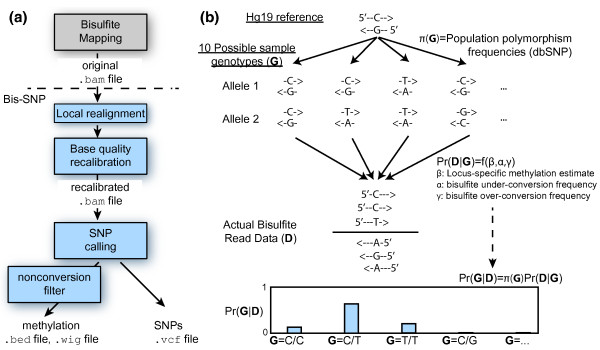
**Bis-SNP workflow**. (**a**) Bis-SNP accepts .bam files, produced by a genome mapping tool (BSMAP, MAQ, Novoalign, Bismark, and so on). The local realignment and base quality recalibration steps result in a new BAM with the recalibrated base quality scores. Finally, Bis-SNP performs SNP calling and outputs both methylation levels and SNP calls. (**b**) The SNP calling step is performed on each genomic position independently. Differences between the reference genome and the sample genome can produce one of 10 possible allele pairs or genotype (**G**, only 4 shown here). Frequencies of all possible substitutions in the population are taken from the dbSNP database and represented as *π*(**G**). A probabilistic model that incorporates prior probabilities for methylation level and bisulfite conversion efficiency is used to calculate the probability of observing the actual bisulfite read data (**D**) assuming each of the 10 genotypes (*Pr*(**G**|**D**)) Finally, bayesian inference uses the population frequencies of each SNP to calculate the posterior likelihood *Pr*(**D**|**G**).

The two primary steps in the Bis-SNP workflow are outlined in Figure 2a and include base quality re-calibration and local realignment followed by SNP calling. Bis-SNP accepts standard alignment files (.bam format), which can be generated by popular Bisulfite-seq mapping programs such as MAQ, Bismark, BSMAP, PASH, or Novoalign (reviewed in [10]). This allows the user to decide which mapping criteria are most important for their specific application. This also makes Bis-SNP compatible with specialized mappers such as RRBSMAP [33] and any other program that can output (.bam) files.

The Bis-SNP model relies on the accuracy of base quality scores, which are initially estimated by the instrument-specific base caller. However, these initial base scores do not accurately represent true error probabilities, which are highly dependent on local sequence context [12]. In the GATK workflow, empirical mismatch rates for each nucleotide at each sequencing cycle are calculated by comparing base calls to the reference genome, and these mismatch rates are used to recalibrate instrument-generated values [12]. We cannot use this default implementation with bisulfite-seq data, because true C>T sequencing errors can not be identified when the underlying methylation state of each bisulfite-converted DNA fragment is unknown. Therefore, instead of treating Ts at reference cytosines as errors, we treat them as a 5th base *X*, and estimate these as a group separately from T>T, A>T, or G>T. The effect is that we can effectively recalibrate base call quality scores for all except the *X *nucleotide, improving our ability to accurately identify SNPs. Importantly, we are able to improve SNP calling at cytosines by recalibrating 'G-strand' Gs that are complementary to the cytosine.

The user can choose among several output files. For methylation levels, Bis-SNP can return a standard UCSC .bed or .wig file, and a separate output file is generated for each cytosine context specified by the user on the command line. Example cytosine contexts are CG, CH, or CHH (H is the IUPAC symbol for A,C, or T). The .wig output contains the methylation percentage for each methylated cytosine, while the .bed format also contains the number of C/T reads the percentage is based on, plus the strand of each cytosine relative to the reference genome. For SNPs, Bis-SNP can return a Variant Calling Format (.vcf) file, which contains all SNP calls and likelihood scores in addition to methylation percentages.

### Description of SNP calling algorithm

The core of the SNP calling algorithm is based on the Bayesian inference model of GATK [12], and implemented using GATK's LocusWalker class. For each locus, Bis-SNP evaluates one of ten possible diploid genotypes (**G**), as shown in Figure 2B (a diploid genotype is made up of two parental alleles, referred to as *A *and *B*). The prior probability of each genotype, *π*(**G**), is determined using population data from dbSNP (including 1000 genomes data) similar to SOAPsnp [13] (See Materials and Methods). In this model, the likelihood of observing all base calls at a particular locus, assuming a particular diploid genotype *AB*, is expressed as *Pr*(**D**|**G **= *AB*) and is the product of observing the base call at each individual read *j *(Equation 2 of Materials and Methods). As described below, *Pr*(*D_j_*|**G **= *AB*) is calculated according to the strand of read *j *and several bisulfite-specific parameters, *β,α *and *γ *(Figure 2b).

In the GATK non-bisulfite SNP calling model, the probability of observing a base call different from the presumed genotype **G **is simply the base call quality score (defined as the probability of a base calling error). In the case of Bisulfite-seq, this is true for A:T genotypes but not C:G. For C:G genotypes, the probability of observing a T depends on the strand of the read, the methylation state, and the efficiency of bisulfite conversion. Reads on the G-strand opposite the cytosine are treated with the normal GATK model. Reads on the C-strand use an alternate model that considers C>T substitutions as either potential errors or bisulfite conversions (see Materials and Methods). The probability of observing a bisulfite conversion event depends on both the underlying methylation state and bisulfite conversion errors. While none of these are observed directly, they are included in the model as variables *β,α *and *γ *as described in Equation 5 in the Methods section.

After bisulfite treatment, an unmethylated C that fails to get converted to a T is referred to as an *underconversion*, while a methylated C that is converted to T is referred to as an *overconversion*. The underconversion rate, *α*, is often estimated using either a spike in control [4] or the unmethylated mitochondrial genome [6]. This rate can be set manually by the user and has a value of 0.25% by default. While bisulfite overconversion can not be reliably measured using current Bisulfite-seq data, we include an additional parameter, *γ*, which is set to 0% by default. In the future, this could be estimated by spiking in fully-methylated control DNA.

The percentage of methylated reads at a given cytosine position can vary widely. Since C reads and T reads yield more information about the presence of a C>T SNP than T reads, the locus-specific methylation rate can strongly influence SNP calling. In mammalian genomes, CpG methylation levels are multimodal, with various classes of functional elements having distinct methylation patterns. At least four different classes exist with mean methylation rates ranging from around 0% to over 80% [4,24]. Furthermore, methylation at particular di- or tri-nucleotide contexts is organism and even cell type specific. To better understand how methylation estimates could affect SNP calling performance, we implemented several different methods for estimating the methylation frequency parameter *β*, which we describe next.

First, we used a *naive *estimate for *β *where the probability of a read being methylated or unmethylated at any particular cytosine position was 0.5. Second, we used *context-specific *estimates which were determined in a two-round procedure as follows. In the first round, *naive *estimates were used as described above, and the resulting SNP calls were used along with dbSNP to select a set of high-confidence non-SNP homozygous cytosines (probability>99.99%). These homozygous cytosines were used to estimate average methylation levels for a set of cytosine sequence contexts that could be specified on the Bis-SNP command line (by default, set to *β_CG _*and *β_CH_*). In the third and final estimation method, *β *was estimated for each cytosine locus individually using the number of C and T reads ($\frac{c}{c+t}$). The rationale for this *locus-specific *method was our concern that genome-wide estimates might be inappropriate CpGs, given the strongly bimodal nature of CpG methylation levels. Each of these three *β *estimation methods was run individually as described below. The default method for the public version of Bis-SNP is *locus-specific *estimation.

### Evaluation of SNP calls at known SNPs

We evaluated Bis-SNP calling accuracy for each of the three different methylation estimation methods (*naive*, *context-specific*, and *locus-specific*). The latter two methods performed substantially better than *naive *estimation, so those are the only two discussed below. We evaluated accuracy using an actual whole-genome Bisulfite-seq dataset from a normal (male) human colon mucosa sample published previously by our lab [6] (sequence available via accession dbGap:phs000385). All reads were 75 bp long single-end, and generated using the Illumina Genome Analyzer IIx platform. The complete dataset had an average read depth of 32X. The Bisulfite-seq data were compared to Illumina Human1M-Duo BeadChip SNP array data from same sample.

**Figure 3:**
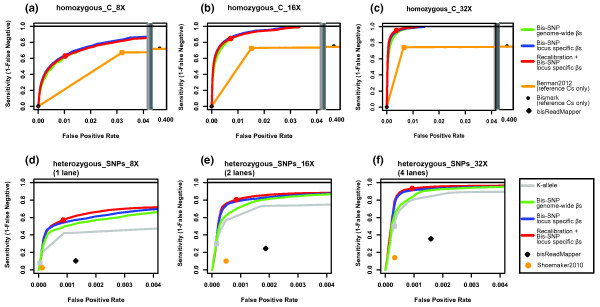
**Bis-SNP error frequencies in detecting SNPs on the Illumina 1 M SNP array**. Receiver Operating Characteristics (ROC curves) are shown for Bis-SNP accuracy at detecting SNPs in Bisulfite-seq data derived from human colonic mucosa tissue. The 'true' genotypes were determined using an Illumina Duo 1 M Human SNP array, and Bis-SNP results were only evaluated at these million genomic positions. All datasets were from [6]. The three ROC curves at the top (a-c) show accuracy at positions corresponding to 435,120 homozygous cytosines on the 1 M SNP array. By randomly downsampling from the average 32× read depth of the Bisulfite-seq data, we are able to show results corresponding to 8× coverage (**a**), 16× coverage (**b**). Bis-SNP using three different conditions is compared to Bismark and the method used in 'Berman2012' [6], both of which restrict their results to reference cytosines. For 'Berman2012', we varied the number of reverse strand G reads required to plot a range of stringencies. The three plots at the bottom (**d-f**) show accuracy at the 303,656 positions that are heterozygous according to the 1 M SNP array. For comparison, we show results from the k-allele method (similar to the approach of [30]), Shoemaker2010 [20] and bisReadMapper[3].

The primary goal of bisulfite sequencing is the accurate determination of cytosine methylation levels, so we first investigated the ability of Bis-SNP to correctly identify homozygous cytosines. As the 'ground truth', we used 435,120 positions identified as homozygous cytosines on the 1 M SNP array, and examined false negative and false positive calls made by Bis-SNP (Figure 3a-c). Calls at varying stringencies were generated by adjusting the Bis-SNP score cutoff, which is defined as the odds ratio between the first and second most likely genotype (see Methods). Evaluating the different Bis-SNP methylation estimates with and without base quality recalibration showed that the *locus-specific β *estimation plus recalibration produced the most accurate results. Using the complete sequence dataset and the default score cutoff (Figure 3c, red circle), Bis-SNP was able to detect 95.22% of the true cytosines (414,327 features) with a false positive rate of 0.37% (2,461 features). We simulated lighter sequencing coverage by randomly picking reads from the full dataset to estimate accuracy at 8× (Figure 3a) and 16× (Figure 3b) genomic coverage. The reader should note that these false positive rates are not indicative of the genome-wide false positive rates, since most false positives come from heterozygous SNPs which are frequent on the SNP array but very infrequent in the genome.

For comparison, we determined the accuracy of homozygous cytosine calling using several published methods (Figure 3a-c). Bismark[34] returns methylation estimates for all cytosines in the reference genome. It is thus not surprising that Bismark performs poorly for features on the 1 M SNP array, which were selected for their polymorphism and differences from the reference genome. Several other published studies use the same strategy and estimate methylation at all reference cytosines [35,36]. In our own earlier work [6], we also restricted methylation calling to reference cytosines. Thus it is not surprising that when we applied this method ('Berman2012') to the 1 M SNP array dataset, it achieved almost the same false negative rate as Bismark. However, 'Berman2012' filtered out positions where less than 90% of reads were C or T on the C-strand and G on the G-strand, resulting in a substantially lower false positive rates than Bismark, but not as low as Bis-SNP.

We next focused on the ability of Bis-SNP to determine heterozygous SNPs, which can be used both for improving methylation calling accuracy as well as allele-specific methylation analysis (see Figure 1b). Heterozygous SNPs are more difficult to identify than homozygous SNPs, due to the approximately 1/2 the read coverage for each allele. We excluded the haploid × chromosome, leaving 303,656 autosomal loci called as heterozygous by the 1 M SNP array. As before, the *locus-specific β *methylation estimation plus recalibration performed the best of all methods. Using the full dataset with the default Bis-SNP cutoff (Figure 3c, red circle), Bis-SNP was able to identify 93.18% of heterozygous SNPs (282,944 loci) with a false positive rate of 0.094% (755 loci). Of the 303,656 heterozygous loci examined, 242,347 (79.81%) were C/T heterozygotes. C>T is the most common SNP in mammals, arising from evolutionary deamination of methylated cytosines. It is also the most difficult SNP to detect in bisulfite-treated DNA, because the C-strand reads are often uninformative (see Figure 1). As expected, Bis-SNP (and other methods) performed more poorly on C/T heterozygous SNPs than others, due to C>T conversion ambiguity (Additional File 2).

We compared Bis-SNP results to heterozygous SNPs called using two alternate 'k-allele' techniques that used read count cutoffs without incorporating base quality scores. We implemented a generalized form of the method used by [21,30] to use a variable read count cutoff. This cutoff, *k*, was defined as the minimum percentage of reads with a secondary allele necessary to call a heterozygous SNP. As in [30], we counted C and T as a single allele at reference cytosines (on the C-strand only). In addition to k-allele, we also tried the Shoemaker method [20], which does not evaluate C/T SNPs at all and requires observations of the less frequent allele on at least 20% of reads on each strand. Finally, we tried the bisReadMapper algorithm [3], which calls SNPs independently on each strand using a non-bisulfite SNP caller, SAMTOOLS [11], and reports only those SNPs that agree between strands. Figures 3d-f show that each variation of Bis-SNP performs better than other methods.

**Figure 4:**
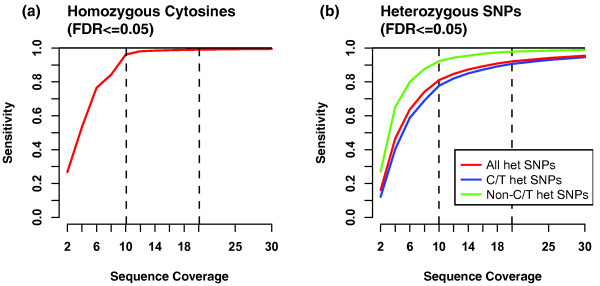
**Sensitivity as a function of sequence coverage**. Comparisons between Bis-SNP SNP calls and 1 M SNP array from Figure 3 ROC curves were extended to a range of coverage levels from 2×-30×. At each coverage level, we selected the least stringent threshold that yielded a False Discovery Rate (FDR) less than 0.05, and plotted the Sensitivity (1 - False Negative rate). As in Figure 3, separate plots show sensitivity at detecting homozygous cytosines (**a**) and heterozygous SNPs (**b**). For heterozygous SNPs, we include the overall detection rate (red line), as well as separate lines for C/T heterozygous SNPs (blue line) and non-C/T heterozygous SNPs (green line).

An important practical question is the minimum read depth required for accurate SNP identification. We addressed this problem by downsampling our 32× Bisulfite-seq genome to various coverage levels from 2× to 30× (Figure 4). For each coverage level, we determined the number of false positives and false negatives across a range of Bis-SNP stringency cutoffs using the 1 M SNP array data, as in Figure 3. At each coverage level, we then selected the least stringent cutoff that produced a False Discovery Rate (FDR) of less than 5%, and plotted the number of true positives (sensitivity). For both homozygous cytosines (Figure 4a) and heterozygous SNPs (Figure 4b), sensitivity increased dramatically up to about 10× coverage and then began to level off. Homozygous SNPs were almost fully detected (98% sensitivity) by 10× coverage, while heterozygous SNPs had a more gradual increase from 80% detected at 10× to 95% detected at 30×.

### Accuracy of genome-wide methylation calling

To verify the ability of Bis-SNP to correctly identify cytosines and improve methylation quantification genome-wide, we ran Bis-SNP across an entire chromosome for the OTB colon mucosa sample and four additional whole-genome bisulfite-seq samples (Table 1). TCGA normal lung and normal breast were generated by the USC Epigenome Center and aligned using BSMAP, while the two mouse methylomes were generated by UCSD and aligned using Novoalign [22]. Runtimes for chromosome 1 were about 3 hours using a standard 12-core Intel server with 10 GB RAM (Intel, Santa Clara, CA, shown). The entire human genome takes about 30-40 hours on a single server (data not shown).

**Table 1:** Chromosome 1 Bis-SNP detection

Sample	Aligner	reference	cvg	Het SNPs	Hom SNPs	Callable bases	runtime
OTB	MAQ	hg18	32×	119,103	67,725	211,042,010	2.8 h

TCGA-lung-normal	BSMAP	hg19	19×	118,412	58,309	222,763,786	3.1 h

TCGA-breast-normal	BSMAP	hg19	19×	113,009	57,281	221,014,965	2.7 h

Mouse-F1i	Novoalign	mm9	50×	663,528	65,364	178,718,615	3.1 h

Mouse-F1r	Novoalign	mm9	41×	682,979	67,068	178,847,508	3.1 h

Notes: All benchmarking performed using a single Intel(R) Xeon (X5650,2.67 GHz) server with 12 CPU cores and 10 GB memory. SE refers to single-end sequencing and PE to paired-end.

**Figure 5:**
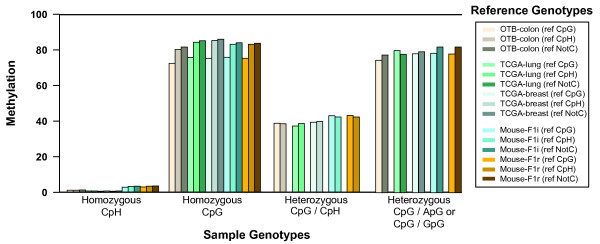
**Accurate methylation calling at SNPs**. Bis-SNP was run on five different datasets, single-end sequencing from Colon Mucosa Tissue [6] (**a**), two TCGA samples using paired-end sequencing from breast and lung tissues (normal, non-cancer), and two mouse samples using paired-end sequencing from [22] (see Table 1). In each case, Bis-SNP was used to identify cytosines in one of four sequence context in the sample genome. For each sample genotype, cytosines were further divided by their sequence context in the reference genome ('ref CpG', 'ref CpH', or 'refNotC'). All cytosines within a particular category in a particular sample were averaged to yield a mean methylation level. The number of cytosines in each category can be found in Table 2.

We used Bis-SNP to identify four classes of cytosines in the sample genome (Figure 5 and Table 2 'Sample Genotypes'), and separated these by their corresponding sequences in the reference genome (Figure 5 and Table 2 'Reference Genotypes'). As shown in Table 2 about 0.5-0.6% of reference CpGs were lost in the sample genome, and 0.5-0.6% of CpGs in the sample genome were lost in the reference. The two mouse samples had significantly higher SNP rates, presumably due to true strain differences between the crossed strains and the C57BL/6J strain sequenced for the mouse reference genome. In both F1 mice, about 2.5% of reference CpGs were lost in the sample genome, and about 1.1% of CpGs in the sample genome were lost in the reference.

**Table 2:** Chromosome 1 cytosine counts and methylation

Sample	Sample genotype	Reference Genotypes						% methylation		
		**Reference CpG**		**Reference CpH**		**Reference DpN (D = A,T,G)**		**Ref CpG**	**Ref CpH**	**Ref DpN**

OTB normal colon	CpG	3,758,803	99.39%	12,540	0.02%	11,838	0.01%	73%	80%	82%
	
	CpH	7,773	0.21%	78,427,918	99.95%	18,804	0.01%	1%	1%	1%
	
	DpN	5,658	0.15%	14,166	0.02%	128,570,817	99.97%	NA	NA	NA
	
	CpG/CpH het	7,218	0.19%	8,998	0.01%	NA	NA	39%	39%	NA
	
	CpG/RpG het	2,512	0.07%	NA	NA	1,826	0.00%	74%	NA	77%

TCGA Normal lung	CpG	4,153,196	99.52%	10,995	0.01%	10,511	0.01%	76%	84%	85%
	
	CpH	5,460	0.13%	85,031,960	99.96%	16,420	0.01%	1%	1%	1%
	
	DpN	5,310	0.13%	13,725	0.02%	133,490,905	99.98%	NA	NA	NA
	
	CpG/CpH het	6,682	0.16%	8,529	0.01%	NA	NA	37%	39%	NA
	
	CpG/RpG het	2,476	0.06%	NA	NA	1,993	0.00%	80%	NA	78%

TCGA normal breast	CpG	4,100,643	99.54%	10,893	0.01%	10,657	0.01%	75%	85%	86%
	
	CpH	5,286	0.13%	80,654,084	99.96%	13,390	0.01%	1%	1%	1%
	
	DpN	4,954	0.12%	13,310	0.02%	136,180,779	99.98%	NA	NA	NA
	
	CpG/CpH het	6,289	0.15%	8,120	0.01%	NA	NA	39%	40%	NA
	
	CpG/RpG het	2,413	0.06%	NA	NA	1,854	0.00%	78%	NA	79%

Xie 2012 Mouse F1i (chr1)	CpG	2,125,320	97.51%	10,990	0.02%	11,757	0.01%	76%	83%	84%
	
	CpH	4,314	0.20%	57,706,841	99.87%	20,312	0.02%	3%	3%	3%
	
	DpN	5,300	0.24%	20,905	0.04%	118,570,097	99.96%	NA	NA	NA
	
	CpG/CpH het	28,896	1.33%	36,735	0.06%	NA	NA	43%	42%	NA
	
	CpG/RpG het	15,754	0.72%	NA	NA	12,917	0.01%	78%	NA	82%

Xie 2012 Mouse F1r (chr1)	CpG	2,199,907	97.52%	11,268	0.02%	11,974	0.01%	75%	83%	84%
	
	CpH	4,476	0.20%	58,685,115	99.87%	20,933	0.02%	3%	3%	4%
	
	DpN	5,171	0.23%	20,765	0.04%	117,647,445	99.96%	NA	NA	NA
	
	CpG/CpH het	29,983	1.33%	38,159	0.06%	NA	NA	43%	42%	NA
	
	CpG/RpG het	16,371	0.73%	NA	NA	13,147	0.01%	78%	NA	82%

Notes: 'het' signifies heterozygous. Two non-reference bases in a row automatically filtered out. CpH = C(A/C/T). DpN = (A/T/G)(A/C/T/G). RpG = (A/G)G. CpG/TpG heterozygous genotypes are filtered out because they can not be used for methylation calling.

We next compared average methylation levels across each sample genotype (Figure 5). As expected, homozygous CpHs were consistently low, while homozygous CpGs were consistently high, regardless of the corresponding reference sequence. Both mouse frontal cortex brain samples showed elevated levels of CpH methylation as described in the original publication [22]. Interestingly, homozygous CpGs that represented SNPs (where the sample differed from the reference genome) had consistently higher methylation. This fits with what is known about mammalian genome evolution - evolutionary C>T changes occur much more frequently at methylated than unmethylated CpGs because the C>T deamination and deamination repair process is methylation-specific. We next looked at heterozygous CpGs (Figure 5, right). CpG/CpH positions had methylation about halfway between CpG homozygous and CpH homozygous positions. At CpG/ApG or CpG/GpG heterozygous positions, methylation can only be measured for the C allele, and the methylation state is about the same as homozygous CpGs. CpG/TpG heterozygous positions are not shown, because we can not accurately measure methylation at these positions. Together, these data show that Bis-SNP genotype calling produces accurate methylation quantification even when the sample genome differs from the reference genome.

## Conclusions

We have described a publicly-available software tool, Bis-SNP, which extracts methylation information and SNP information simultaneously from data generated using the Illumina Bisulfite-seq protocol. Command-line executables (Additional File 3) and open-source code (Additional File 4) are both freely available for download [37]. The directional nature of the Illumina protocol allows for analysis of DNA methylation and the identification of a SNP at the same position, by combining information from each strand separately. This is the dominant Bisulfite-sequencing protocol in use today by individual labs and genomics consortia such as ENCODE, the NIH Epigenomics Roadmap, and The Cancer Genome Atlas. By correctly identifying and filtering SNPs correctly, we can obtain more accurate methylation levels and heterozygous SNPs, including C/T SNPs, can be used to identify allele-specific methylation patterns. Bis-SNP is implemented using the efficient GATK framework, which allows for runtimes that are reasonable for modern whole-genome analysis. An entire 32× whole-genome dataset took about 30 hours to run on a typical 12-processor compute node with 10 GB of memory, or 3 hours when each chromosome was run in parallel on a separate compute node. This performance profile makes Bis-SNP accessible to most users.

We included the capability to perform base quality re-calibration on bisulfite-seq data, which improves the overall SNP calling accuracy of Bis-SNP. Not only do more accurate base quality scores allow us better identification of SNPs as shown here, but could be used in the future to calculate more precise DNA methylation estimates. Biological DNA samples do not typically have a large number of cytosines that are always 100% methylated, so there is not a reliable way to identify true C>T mismatches and recalibrate quality scores at these positions. Recalibration could be improved in the future by spiking a library of DNA that has not been treated with bisulfite into the same sequencing lane.

The potential applications of Bisulfite-seq in basic biology and medicine are broad, and Bis-SNP can be used for the majority of Bisulfite-seq experimental designs including Whole-Genome Bisulfite-Seq (WGBS), Reduced Representation Bisulfite-Seq (RRBS), and customizable genome selection methods. While we have focused on human studies, Bis-SNP can output methylation levels split up according to user-defined cytosine contexts, which makes it applicable to analysis of *Arabidopsis *or any other organism. It also allows Bis-SNP to accommodate novel study designs, such as *in vitro *methylation by methyltransferases with arbitrary sequence specificities, or even the study 5-hydromethyl-cytosine (5-hmC) using a novel bisulfite-sequencing approach [38].

An intriguing potential use of Bisulfite-seq and Bis-SNP is the study of genome-wide associations between SNPs and DNA methylation patterns (i.e. *methQTLs*, reviewed in [39]). While the experimental designs thus far have envisioned paired SNP and methylation assays, our encouraging results with Bis-SNP suggest that both could be captured in a single Bisulfite-sequencing experiment. Sequencing depths of 50× or greater for Whole-genome Bisulfite-seq are not unattainable from a cost perspective, and would likely provide sufficient SNP and methylation coverage for methQTL studies. Another potential application could be a Genome-Wide Association Study (GWAS) that uses Bisulfite-seq rather than traditional sequencing, to identify disease associations at the genetic and epigenetic levels simultaneously. This could be especially useful given the large number of GWAS hits that appear to affect regulatory regions rather than gene coding regions. Bis-SNP and other Bisulfite-seq analysis tools will be important in the development of these exciting new technologies.

## Materials and methods

### Local realignment, base quality recalibration and other BAM file preprocessing

Reads with mapping quality scores less than 30 and those mapped to multiple genomic regions were removed, as are PCR duplicates (optional). For paired-end reads, we remove read pairs that do not have the ProperlyPaired field set.

We use GATK to perform local multiple sequence realignment and sequence recalibration mostly as described [12]. Since most of bisulfite sequencing mapping tools (e.g. Bismark, BSMAP, MAQ etc) do not provide correct CIGAR string in the BAM file for GATK's indel realignment, the CIGAR string is recalculated when necessary. We extend GATK's RealignerTargetCreator to count mismatch number but not count thymine as a mismatch when the reference genome position is cytosine. After we create a potential indel interval, we realign using a modified version of GATK's IndelRealigner. PCR duplicate reads are marked after indel realignment.

For base quality recalibration, we modify the GATK algorithm to account for bisulfite conversion by extending the GATK CountVariantWalker and TableRecalibrationWalker classes. The algorithm first tabulates empirical mismatches to the reference at all loci not known to vary in the population (i.e., not in dbSNP build 135). These counts are categorized by their reported instrument-reported quality score (*R*) and position (cycle) within the read (*C*). In tabulating mismatches, we do not count thymine as a mismatch when the reference genome position is cytosine (on the second end of a paired-end read, we instead don't count adenine as a mismatch when the reference is guanine).

By default, only positions with a recalibrated Base Calling Quality Score of greater than 5 are used for SNP calling. This quality cutoff can be set using a command line parameter (see User Manual in Additional File 3).

### BisSNP probabilistic model

We begin with the bayesian likelihood model of GATK ([12]), and make a number of bisulfite-specific adaptations. Assuming the underlying genome is diploid, we let **D **= (*D*_1_, *D*_2_, ..., *D_r_*) represent the base calls at a particular genomic position *i *that is covered by *r *sequencing reads. We then calculate the posterior probability by (1) as in GATK:

(1) $$Pr\left(G|D\right)=\frac{\pi \left(G\right)Pr\left(D|G\right)}{Pr\left(D\right)}$$

Here, *G *is the underlying diploid genotype, *AB*, with *A *and *B *being the two parental alleles. *π*(*G*) is a genotype prior probability for observing the given genotype based on the genotype of the reference genome and population frequencies, the same as discussed in Table 1 of SOAPsnp paper [13]. *Pr*(*D*) is defined as the sum over all possible genotypes ∑*_AB _π*(*AB*) *Pr *(**D**|*AB*), but is the same in each case and can generally be ignored since we are concerned with likelihood ratios. We assume that each of the two alleles are equally likely to be sequenced, and calculate the overall likelihood of **D **as the product of all individual reads (2),(3):

(2) $$Pr\left(D|G\right)=\overset{r}{\underset{j=1}{\prod }}Pr\left(D_{j}|G\right)$$

(3) $$Pr\left(D_{j}|G=AB\right)=\frac{1}{2}Pr\left(D_{j}|A\right)+\frac{1}{2}Pr\left(D_{j}|B\right)$$

The following steps are shown for single-end sequences. For paired end sequences, the first end is treated as described, but the second end is reverse complemented before performing these calculations (because the Illumina second end is the complementary strand of the same template as the first end). This changes G>A bisulfite substitutions, which occur on the second end, to the actual C>T substitutions present on the bisulfite-converted template. The recalibrated base quality scores are on a phred scale which represents the probability *ε *that the position is an error, which is used in the following calculation.

When the underlying allele is adenine (a), thymine (t), bisulfite conversion does not apply and the probability estimation is straightforward as shown for t:

(4) $$\text{Pr}\left(D_{j}|B=\text{t}\right)=\left\{\begin{array}{cc} \frac{\varepsilon_{j}}{3} & \;\text{if}\;D_{j}\neq \text{t}\\ 1-\varepsilon_{j} & \;\text{if}\;D_{j}=\text{t} \end{array}\right)$$

Here, *ε_j _*is the probability of a sequencing or base calling error at position *j*, i.e. probability that the true allele *B *is a t, but base call *D_j _*is observed as an a, c, or g. The likelihood function for a is equivalent to that of Equation (4). When the underlying allele is a c or a g, however, the probabilities are strand-specific since bisulfite conversion only affects one strand in the directional Bisulfite-seq protocol (Figure 1). The probability of seeing a t in the read depends on the probability that the position is methylated (*β*), as well as the bisulfite conversion efficiency (*α *and *γ*). Bisulfite treatment converts all unmethylated cytosines to thymine, but in practice it is not 100% efficient [4]. The parameter *α *is the estimated frequency of unmethylated cytosines which are not converted (typically taken from unmethylated spiked in DNA [4] or the mammalian mitochondrial sequences, which we have found to be almost completely unmethylated [6]. In this case, *α *= *β_chr_M*). By default, *α *is set to 0.0025 but can be specified by the user. We also include a *γ *parameter for *over-conversion*, i.e. the rate at which methylated cytosines are converted. Although this is not routinely measured in practice, it could be estimated by including an enzymatically methylated control DNA [40], or a sequencing library without bisulfite conversion. By default, *γ *is set to 0 but can be specified by the user. The full likelihood calculation for cytosines is as follows:

(5) $$\begin{array}{c} Pr\left(D_{j}|B=\text{c}\right)=\left\{\begin{array}{cc} \left(1-\varepsilon_{j}\right)\left[\beta_{j}\left(1-\gamma \right)+\left(1-\beta_{j}\right)\alpha \right] & \;\text{if}\;D_{j}={\text{c}}^{+}\\ \frac{\varepsilon_{j}}{3}+\left(1-\varepsilon_{j}\right)\left[\beta_{j}\gamma +\left(1-\beta_{j}\right)\left(1-\alpha \right)\right] & \;\text{if}\;D_{j}={\text{t}}^{+}\\ 1-\varepsilon_{j} & \;\text{if}\;D_{j}={\text{c}}^{-}\\ \frac{\varepsilon_{j}}{3} & \;\text{otherwise} \end{array}\right)\\ \beta_{j}\left(\text{1}-\gamma \right)=\text{methylated and }\left(\text{properly}\right)\text{ not converted}\\ \beta_{j}\gamma =\text{methylated and }\left(\text{improperly}\right)\text{ converted}\\ \left(1-\beta_{j}\right)\alpha =\text{unmethylated and }\left(\text{improperly}\right)\text{ not converted}\\ \left(\text{1}-\beta_{j}\right)\left(\text{1}-\alpha \right)=\text{unmethylated and }\left(\text{properly}\right)\text{ converted} \end{array}$$

The key to these calculations is that reads on the same strand as the inferred cytosine allele (denoted with +) are treated differently than reads from the opposite strand (denoted with -). As expected based on the example in Figure 1, a true allele of *B *= c results in a very high probability of seeing a t^+ ^(a 't' read on the C-strand), but a very low probability of seeing a t^- ^(an 'a' read on the G-strand). The genotype *G_best _*with the highest posterior probability *Pr*(*G*|**D**) is chosen, and the final output score is the odds ratio between the best (*G_best_*) and the second best (*G_nextbest_*), as in Equation (6). In practice, we optimize execution by evaluating only the subset of the 10 possible diploid genotypes that are possible given the sequences read.

(6) $$score=log(\frac{Pr\left(G_{best}|D\right)}{Pr\left(G_{nextbest}|D\right)}$$

Bisulfite efficiency, i.e. *α *and *γ *typically vary by less than 1%, so the critical parameter included in Equation 5 is the methylation rate *β*. Since this rate varies by genomic context, organism, and even cell type, we allow the user to specify the possible contexts as a set of *n *nucleotides sequences specified by their IUPAC degeneracy codes (for instance, *CH *represents *CC*, *CT*, or *CA*). In mammalian genomes where typically only the single base 3' of the cytosine is considered relevant, the user would specify CG and CH (the Bis-SNP default). For *Arabidopsis*, one might specify CG, CHH, and CHG. Any arbitrary number of 5' and 3' bases may be specified in order to accommodate the full range of Bisulfite-seq assays. For instance a CCGG pattern could be specified for MspI restriction sites inherent to the RRBS protocol ( [41]).

One methylation output file (BED6+2 format) is created for each cytosine context specified by the user. For each cytosine determined to have the particular sequence context, the percent methylated (the number of C reads on the C-strand divided by the number of C or T reads on the C-strand) is output as the score field. To aid in statistical analysis, a second field contains the total number of C/T reads.

### Five-prime bisulfite non-conversion filter

Non-conversion of unmethylated Cs is known to preferentially affect the 5' end of Illumina-generated reads, most likely driven by the re-annealing of sequences adjacent to the fully methylated sequence adapters during bisulfite conversion. We control for this using a 5' non-conversion filter as implemented in our earlier work [6]. For each read, we walk along the read from 5' to 3', and we remove any Cs on the C-strand until we reach the first reference C which is converted to a T. By applying this filter, early bisulfite conversion in early cycles is brought to levels very similar to those of late cycles, thus removing a potential source of methylation bias (data not shown). Notice that this filter should be turned off for RRBS data, which gleans most of its methylation data from the first cycle (see user manual).

### Pre-SNP calling quality filters

Using the approach of GATK, we apply additional quality filters before SNP calling to avoid known sources of false positives. SNPs found in clusters (two or more within a ten-base-pair window) were filtered out. SNPs with coverage depth above 120, Strand Bias(SB) score more than -0.02, or Quality by Depth(QD) less than 1.0 are filtered out. All of these parameters are configurable (see User Manual). If BAM contains Mapping Quality scores, suspicious regions are filtered out when greater than 10% of aligned reads (minimum of 40 reads) have mapping quality of 0.

Bisulfite sequencing can have higher strand biases since high bisulfite concentration can lead to DNA degradation when the depurination step causes random strand breaks [42,43]. We calculated strand bias score as in GATK, but bisulfite converted reads have an apparent strand bias which is higher than the actual strand bias, since the G-strand contributes more than the C-strand at cytosines. For this reason, we used a substantially less stringent strand bias cutoff (-0.02) than the GATK default.

### Downsampling coverage

We downsampled the human colon mucosa Bisulfite-seq dataset into different mean coverages using GATK, which randomly picks *z *reads at each individual nucleotide locus. The following formula is used, where *N *is the mean coverage of total dataset before downsampling (32× in this case), *n *is the desired downsampling coverage, and *m *is the actual coverage at the particular locus.

(7) $$z=\frac{m*n}{N}$$

### External tools used for comparison

#### K-allele method

The K-allele method was used to identify heterozygous SNPs as a generalization of described methods [21,30], both of which count the number of alternate alleles present and exclude C/T SNPs. For reference cytosine positions, we only use counts from the *G-strand*, while at other positions we combine the two strands to get read counts. After these filters, we use a *K *cutoff which can vary from 0-10 and apply the *K*-allele threshold as follows. For positions with *n *passing reads where *n *is less than 10, we require that each of the two alleles have at least *K *reads. For positions where *n *is greater than 10, we require at least $n\frac{k}{10}$ reads. Fore reference, the Hudson Alpha group [21] used a set definition *K *of 7 reads and at least 10%, and excluded all C/T SNPs. The UCLA group [30] specified that the allele with the lower read count had to contain at least 40% of reads, and excluded C/T reads.

#### bisReadMapper

We downloaded bisReadMapper version 1 [3]. We first use genomePrep.pl to preprocess the reference genome and extract cytosine position in each chromosome. The built in read mapper could not handle our large BAM file, so we circumvented the mapping step and used the BAM files directly as input. This is not a standard part of the bisReadMapper package, and required us to divide our BAM alignment files to separate reads aligning to the forward strand of the reference genome from those aligning to the reverse strand. We used the following bisReadMapper parameters: allC=1; length=75; snp=dbsnp135.rod; alignMode=S; qualBase=33; trim3=0; trim5=0; refDir=/path/to/GenomePreparationProcessedDir/

#### Shoemaker

The Shoemaker [20] method was implemented as described in their supplemental materials with clarifications from the author. The reads are handled differently based on the ratio of C to T nucleotides within the read and the ratio of G to A nucleotides (if C to T ratio was higher, it was considered a bisulfite-converted C-strand read, otherwise it was considered a complementary read from the 2nd end and it was reverse complemented). All reads are then demethylated *in silico *(Cs converted to Ts). Input reads are filtered by their criteria: (1) Base calls at the examined SNP site and three flanking positions on either side needed to have a minimum Base Quality score of 15. (2) If a certain base was present in more than 20% of reads on one strand, its reverse complement needed to be present on at least 20% of the reads on the opposing strand. Only positions passing these two criteria were analyzed. Base Quality scores were used to weight the nucleotide count contributions to the nucleotide frequency matrix. This matrix was normalized, multiplied by the read count to get final nucleotide number matrix in each location (normalized and weighted A,C,G,T number in each loci). The Fisher exact test was applied to each nucleotide in each of the alleles (e.g. nucleotide number of G vs. nucleotide number of not G, expected nucleotide number of G vs. expected nucleotide number of not G). Two p-values of each allele were multiplied together for each of ten possible genotypes and then normalized. The SNPs were selected out when (1) The best genotype was 10 times more than the next most likely genotype, (2) the SNP was in reported in dbSNP, and (3) had at least 10× read depth.

#### Bismark

We downloaded Bismark-0.50 [34]. We converted our input BAM file to SAM format and ran genome_methylation_bismark2bedGraph.pl to extract cytosines. Default settings were used.

#### Berman2012

We implemented a generalized version of the method described in our earlier work [6]. We only included reference cytosine positions that had at least 3 overlapping C or T reads. We required at least *k*% of reads on the C-strand to be C or T, and *k*% of the reads on the G-strand to be G. The default setting (used in [6] and shown as an orange rectangle in Figure 3) was *k *= 10%.

### Datasets used for whole-genome comparisons

#### OTB-colon

75 bp Single End Whole-Genome Bisulfite-Seq data from [6] was generated using Illumina GAIIx sequencing (available at dbGap:phs000385). Sample was normal adjacent colon mucosa from a male colon cancer patient.

#### TCGA-lung and TCGA-breast

100 bp Paired End Whole Genome Bisulfite-Seq (WGBS) data generated at USC by the TCGA (The Cancer Genome Atlas) USC-JHU Epigenome Characterization Center. Data is unpublished, but available for download via the UCSC Cancer Genomics Hub (CG-Hub [44]). The lung normal sample is adjacent tissue from case TCGA-60-2722 (data available in CG-Hub analysis ID 964a8130-d061-472f-9839-9c1f07b24205), and the breast normal sample is adjacent tissue from case TCGA-A7-A0CE (CG-Hub analysis ID 279507dd-4c62-4975-877d-5cfebd2e7c6f.

#### Mouse-F1i and Mouse-F1r

One hundred-base pair paired-end sequence datasets from two independent mouse samples were used[22]. We downloaded alignments from the original publication (GEO accessions GSM753569 and GSM753570), which were performed using Novoalign. High-confidence genotypes were available for both parental strains via the Mouse Genome Database. We inferred high-confidence genotypes for the progeny only when each parent was homozygous at the particular position.

## Abbreviations

CpG: dinucleotide sequencing consisting of a cytosine followed by guanine; CpH: cytosine followed by an H nucleotide (H is one of C, A, or T); SNP: Single-nucleotide polymorphisms; WGBS: Whole-Genome Bisulfite-Seq; RRBS: Reduced Representation Bisulfite Sequencing; BSPP: Bisulfite Padlock Probes; ENCODE: ENCyclopedia Of DNA Elements; TCGA: The Cancer Genome Atlas; GATK: Genome Analysis Toolkit; VCF: Variant Calling Format; FDR: False Discovery Rate; IUPAC: International Union of Pure and Applied Chemistry; GWAS: Genome-Wide Association Study; BAM: Binary version of the Sequence Alignment/Map (SAM) format; SB: Strand Bias; QD: Quality by Depth.

## Competing interests

The authors declare that they have no competing interests.

## Authors' contributions

YL, PWL, and BPB conceived and designed the study. YL and BPB conceived the statistical approach with input from KDS. YL implemented Bis-SNP and all other computational tools. BPB and YL wrote the manuscript, with input from KS and PWL. All authors have read and approved the manuscript for publication.

## Supplementary Material

Additional file 1 **Detecting heterozygous C/T single nucleotide polymorphisms from Bisulfite-seq data**. Hypothetical bisulfite-seq data with all labels as in Figure 1. This illustrates detection of a C/T heterozygous position (left), and that the G-strand alleles can be used to associate methylation state of an adjacent cytosine on the opposite strand with two parental alleles. Click here for file

Additional file 2 **Bis-SNP error frequencies at C:T heterozygous SNPs**. The data for heterozygous SNP calling in Figure 3c is broken up into C:T SNPs vs. other heterozygous SNPs. Click here for file

Additional file 3 **Bis-SNP executable, utility scripts, and User Manual**. We suggest that the user download the most recent version of these files directly from [37]. Click here for file

Additional file 4 **Bis-SNP source code**. We suggest that the user download the most recent version of these files directly from [37]. Click here for file
