# Supplementary figures and images for: Bis-SNP: Combined DNA methylation and SNP calling for Bisulfite-seq data
Source: Genome Biol. 2012 Jul 11;13(7):R61. doi: 10.1186/gb-2012-13-7-r61 (PMC3491382; doi:10.1186/gb-2012-13-7-r61)

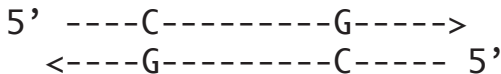

C>T SNP

(het)

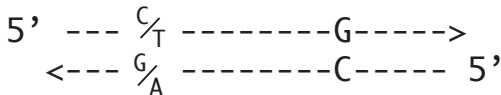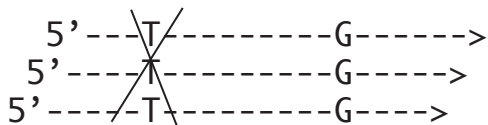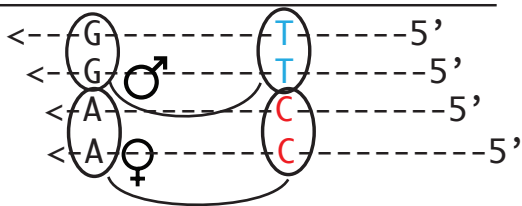

Supplement: Additional file 1 — Detecting heterozygous C/T single nucleotide polymorphisms from Bisulfite-seq data. Hypothetical bisulfite-seq data with all labels as in Figure 1. This illustrates detection of a C/T heterozygous position (left), and that the G-strand alleles can be used to associate methylation state of an adjacent cytosine on the opposite strand with two parental alleles. [file gb-2012-13-7-r61-S1.PDF]

# C/T SNP & Non C/T heterozygous SNPs 32x sequencing coverage

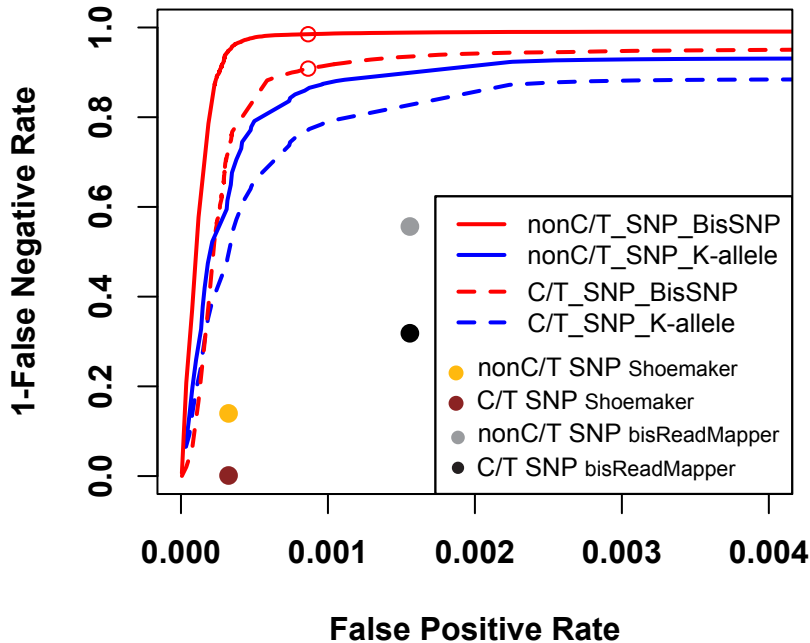

Supplement: Additional file 2 — Bis-SNP error frequencies at C:T heterozygous SNPs. The data for heterozygous SNP calling in Figure 3c is broken up into C:T SNPs vs. other heterozygous SNPs. [file gb-2012-13-7-r61-S2.PDF]
