# Supplementary material for: Bis-SNP: Combined DNA methylation and SNP calling for Bisulfite-seq data
Source: Genome Biol. 2012 Jul 11;13(7):R61. doi: 10.1186/gb-2012-13-7-r61 (PMC3491382; doi:10.1186/gb-2012-13-7-r61)
Supplement: Additional file 3 — Bis-SNP executable, utility scripts, and User Manual. We suggest that the user download the most recent version of these files directly from [37]. [file gb-2012-13-7-r61-S3.GZ › ./Additional_File_3/BisSNP-UserGuide-v0.71.pdf]

Bis-SNP User Guide, v0.71

Yaping Liu, Benjamin P. Berman

2nd July 2012

Bis-SNP is the public available free software (GPL v3 license) for genotyping in bisulfite treated massively parallel sequencing (whole genome Bisulfite-seq(BS-seq), NOMe-seq and RRBS) on Illumina platform. It works for both of single-end and paired-end reads in directional BS-Seq library. It is implemented in Java and based on GATK map-reduce framework for the parallel computation. Copyright belongs to USC Epigenome Center.

1 Prerequisition

1. System: Java(TM) SE Runtime Environment 1.6 (Linux, Mac OSX or Windows).

2 Quick Start

2.1 Download BisSNP program.

Download jar file from `https://sourceforge.net/projects/bissnp/files/`

2.2 Download input files for test

All of the input example file could be downloaded from our website: `http://epigenome.usc.edu/publicationdata/bissnp2011/easyUsage.html` and `http://epigenome.usc.edu/publicationdata/bissnp2011/utilies.html`. All *.bz2 files need to be unzipped firstly.

1. Reference genome file: hg18_unmasked.plusContam.fa.bz2 for hg18, hg19_rCRSchrm.fa.bz2 for hg19.
2. dbSNP file: dbsnp_135.hg18.sort.vcf.bz2 for hg18, dbsnp_135.hg19.sort.vcf.bz2 for hg19.
3. Interval file: I provide whole_genome_interval_list.hg18.bed for the whole genome genotype calling in hg18 and whole_genome_interval_list.hg19.bed for the whole genome genotype calling in hg19.
4. BAM file: normalMerge_chr11-7M-9M.nodups.withhead.bam is BAM file for test. You also need to download normalMerge_chr11-7M-9M.nodups.withhead.bam.bai for BAM file's index or made it from SAMTOOLS by yourself. Since newest GATK no longer support BAM file without ReadGroup tag, BisSNP also require that

input BAM file owns ReadGroup tag. If your own BAM file do not have ReadGroup tag, you should use AddOrReplaceReadGroups.jar in Picard.

2.3 Run BisSNP program in terminal.

1. The following command would output all of the CpG loci in cpg.raw.vcf, all of the SNP loci in snp.raw.vcf, and generate cytosine methylation summary file (.txt):
   java -Xmx4g -jar BisSNP-0.71.jar -R hg18_unmasked.plusContam.fa -T BisulfiteGenotyper -I normalMerge_chr11-7M-9M.nodups.withhead.bam -D dbsnp_135.hg18.sort.vcf -vfn1 cpg.raw.vcf -vfn2 snp.raw.vcf -L chr11:7000000-7100000

3 Usage Detail

BisSNP should be executed from the command line (Terminal, in Linux/UNIX/Mac OSX, or Command Prompt in MS Windows).

java -Xmx4g -jar BisSNP-0.71.jar [options]

3.1 Analysis mode options

-T or --analysis_type < analysis_type >

Type of analysis to run (default option in Bis-SNP is: BisulfiteGenotyper). *Required*.

-C or --cytosine_contexts_acquired < cytosine_context >

Cytosine context to be analyze.(CpG, CpH et .al/). e.g. -C CG,1 CG is the methylation pattern to check, 1 is the C's position in CG pattern. You could specify '-C' multiple times for different cytosine pattern, like: -C CG,1 -C CHH,1 -C CHG... If not specify any pattern, the program would just measure CpG, CpH methylation level by default.

-sm or --sequencing_mode < mode_of_sequencing >

Run BisSNP in different sequencing environment. Bisulfite-seq mode: BM; NOMe-seq mode: GM; Normal non-Bisulfite sequencing mode: NM. (Default is BM)

-out_modes or --output_modes < output_modes >

What kind of output file do you want (options: EMIT_ALL_SITES (emit all of callable sites into vcf file), EMIT_ALL_CONFIDENT_SITES (emit all of sites above emit confidant threshold into vcf file), EMIT_VARIANTS_ONLY (emit all of SNP sites above emit threshold into vcf file), EMIT_ALL_CPG (emit all of CpG sites above emit threshold into vcf file), EMIT_ALL_CYTOSINES (emit all of Cytosine sites above emit threshold into vcf file), EMIT_HET_SNPS_ONLY (emit all of Heterozygous SNP sites above emit threshold into vcf file), EMIT_VARIANT_AND_CYTOSINES(emit all of Cytosine sites above emit threshold into vcf1 file, and all of SNP sites above emit threshold into vcf2 file), DEFAULT_FOR_TCGA (emit all of CpG

sites above emit threshold into vcf1 file, and all of SNP sites above emit threshold into vcf2 file)). Default is DEFAULT_FOR_TCGA, and -vfn2 option is required. (Default is DEFAULT_FOR_TCGA)

-nt or --num_threads < **number_of_threads** >

Enable genotyping in parallel mode and define how many threads should be allocated to running. (Default is single thread)

3.2 Input options

-I or --input_file < **input_file_name** >

input SAM or BAM file(s) for genotyping. *Required*.

-D or --dbsnp < **dbsnp_file_name** >

dbsnp VCF file (which should sort as the same order of chromosome in BAM file header), provide prior SNP information from dbSNP database.You could download it from our website: dbsnp_135.hg18.sort.vcf.gz for hg18, dbsnp_135.hg19.sort.vcf.gz for hg19. *Strongly Recommend*

-R or --reference_sequence < **reference_sequence_file_name** >

Reference sequence fasta file (which should have the same number of contigs as that in input BAM file header). *Required*.

-L or --intervals < **intervals_file_name** >

A list of genomic intervals over which to operate. Can be explicitly specified on the command line like '-L chr11:7000000-7100000', '-L chr1' or in a bed file. I already provided whole_genome_interval_list.hg18.bed for the whole genome genotype calling in hg18 and whole_genome_interval_list.hg19.bed for the whole genome genotype calling in hg19. If not provided, program will go over all the region specified in BAM's header.

3.3 Output options

-vfn1 or --vcf_file_name_1 < **output_vcf_file_name** >

output VCF file, when output mode is DEFAULT_FOR_TCGA, this option is used to store all CpG sites. While -vnf2 option is used to store all SNP sites. And -vnf2 option is required at that time. *Required*.

-vfn2 or --vcf_file_name_2 < **output_vcf_file_name** >

output VCF file, when output mode is DEFAULT_FOR_TCGA, this option is required and used to store all SNP sites. In the other output mode, it is not required. *Conditional Required*

-cpgreads,--file_name_output_cpg_reads_detail < **file_name_output_detailed_cpg_reads** >

Output Haplotype CpG reads bed file that contain each CpG's position, methylation and reads name info. (Default is not enabled)

3.4 Different threshold options

-stand_call_conf < standard_min_confidence_threshold_for_calling >

The minimum phred-scaled threshold for genotype calling that is confident (which is marked PASS in VCF file). (Default is 30)

-stand_emit_conf < standard_min_confidence_threshold_for_emitting >

The minimum phred-scaled threshold for genotype calling that could be emitted. (Default is 0)

-hets,--heterozygosity < heterozygosity_rate >

Heterozygosity value used to compute prior likelihoods for any locus. (Default is 0.001)

-bsRate,--bisulfite_conversion_rate < bisulfite_conversion_rate >

Cytosine bisulfite conversion rate used to compute raw and prior likelihoods for any locus. (Default is 0.9975)

-overRate,--over_conversion_rate < over_conversion_rate >

Cytosine bisulfite over conversion rate used to compute raw and prior likelihoods for any locus. (Default is 0.0)

-vdh,--validateDbsnphet < validate_DbSNP_heterozygousity_rate >

heterozygous SNP rate when the loci is discovered as validate SNP in dbSNP. (Default is 0.1)

-ndh,--novelDbsnpHet < novel_DbSNP_heterozygousity_rate >

heterozygous SNP rate when the loci is discovered as not validate SNP in dbSNP. (Default is 0.02)

-mmq,--min_mapping_quality_score < min_mapping_quality_score >

Minimum read mapping quality required to consider a read for calling. (Default is 30)

-mbq,--min_base_quality_score < min_base_quality_score >

Minimum base mapping quality required to consider a base for calling. (Default is 17)

-toCoverage,--maximum_read_cov < maximum_read_cov >

maximum read coverage allowed. (Default is 250)

-bad_mates,--use_reads_with_bad_mates

if in paired-end mode, allow bad mates that are mapped excessively far away. (Default is not enabled)

-useBAQ,--use_baq_for_calculation

use BAQ for genotype calculation. (Default is not enabled)

-mm40,--max_mismatches_in_40bp_window < maximum_mismatches_in_40bp_window >

Maximum number of mismatches within a 40 bp window (20bp on either side) around the target position for a read to be used for calling. (Default is 3)

-rge,--reference_genome_error < reference_genome_error_rate >

Reference genome error, the default value is human genome, in hg16 it is 99.99% accurate, in hg17/hg18/hg19, it is less than 1e-4 (USCS genome browser described); We define it here default for human genome assembly(hg18,h19) to be 1e-6 as GATK did. (Default is 1e-6)

-tvt,--ti_vs_tv < Transition_rate vs. Transversion_rate >

Transition rate vs. Transversion rate used to compute prior likelihoods for any locus. (Default is 2)

-trim5,--trim_5_end_bp < number_of_5_end_bases_disregarded >

how many bases at 5'end of the reads are discarded. (Default is 0)

-trim3,--trim_3_end_bp < number_of_3_end_bases_disregarded >

how many bases at 3'end of the reads are discarded. (Default is 0)

-minConv,--minmum_cytosine_converted < minmum_number_of_cytosine_converted >

Disregard first few cytosines in the reads which may come from uncomplete bisulfite conversion in the first few cytosines of the reads, still in test yet. (Default is 0)

3.5 Other options for debug only or still in test

-h or --help

Generate this help message

-orad,--output_reads_after_downsampling

Generate Bam file in different sequencing coverage you wanted by downsapling, for performance test only.

-reads,--file_name_output_reads_after_downsampling < output_bam_filename >

Output Bam file's name in different sequencing coverage you wanted by downsapling, for performance test only.

-orcad,--output_reads_coverage_after_downsampling < the_sequence_coverage_you_want >

Output Bam file's mean sequence coverage you wanted after downsapling, for performance test only.

-loc,--test_location < test_location >

output verbose information in likelihood calculation process, only used in debug.

-fnovd,--file_name_output_verbose_detail < file_name_output_verbose_detail >

Output file's name that contain verbose information, if not defined, then information will go to standard error stream, for test only.

-ovd,--output_verbose_detail

Enable to output verbose information, for debug only.

-bcm,--bisulfite_conversion_only_on_one_strand

true: Directional bisulfite-seq protocol which is often used, only bisulfite conversion strand is kept; false: Undirectional bisulfite-seq protocol, which both of two strands are kept, still in test yet.

4 Step by step genotyping tutorial

Here is the basic pipeline for doing Bisulfite-seq genotyping and methylation calling.

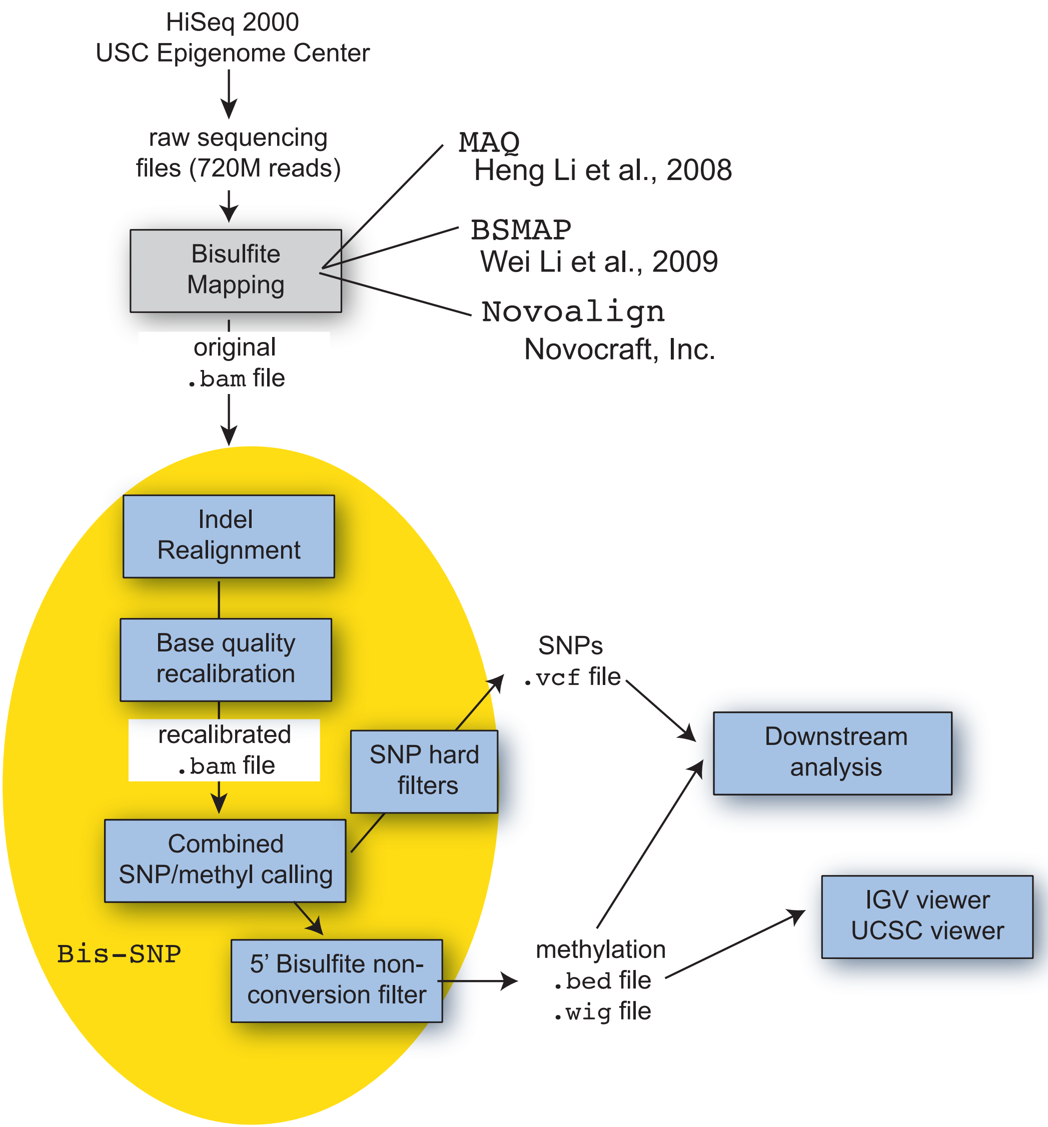

4.1 Add read group tag to BAM file

When your own BAM file do not contained Read group tag. Download Picard tools, then using the following command to add Read group tag to BAM file.

java -Xmx4g -jar AddOrReplaceReadGroups.jar I=sample.withoutRG.bam O=sample.withRG.bam ID=readGroup_name LB=readGroup_name PL=illumina PU=run SM=sample_name CREATE_INDEX=true VALIDATION_STRINGENCY=SILENT SORT_ORDER=coordinate

4.2 Indel realignment

Most of Bisulfite-seq mapping tools do not allow gap alignment yet, they could not find out indels which would cause some of false discovered SNPs. We enable Indel alignment tools on Bisulfite-seq by two steps.

4.2.1 Find indel region

Download known indel files from our website or use your own indel files. Use the following command:

java -Xmx4g -jar BisSNP-0.71.jar -R referenceGenome.fa -I sample.withRG.bam -T BisulfiteRealignerTargetCreator -L region.bed -known indel_1.vcf -known indel_2.vcf -o indel_target_interval.bed -nt cpu_cores

4.2.2 Realign in the indel region

Use the indel_target_interval.bed file generated above to do indel realignment:

java -Xmx4g -jar BisSNP-0.71.jar -R referenceGenome.fa -I sample.withRG.bam -T BisulfiteIndelRealigner -targetIntervals indel_target_interval.bed -known indel_1.vcf -known indel_2.vcf -compress 5 -cigar -o sample.withRG.realigned.bam

4.3 Mark duplicated reads

Use Picard tools to mark the duplicated reads which are mostly come from PCR duplication:

java -Xmx4g -jar MarkDuplicates.jar I=sample.withRG.realigned.bam O=sample.withRG.realigned.mdups.bam METRICS_FILE=sample.withRG.realigned.metric.txt CREATE_INDEX=true VALIDATION_STRINGENCY=SILENT

4.4 Base quality recalibration

BisSNP heavily depend on base quality score for the genotype probability calculation, but Illumina sequencing reads' raw base quality score could not truly reflect the error rate of the base. So we enable the base quality score recalibration for Bisulfie-seq.There are 3 steps to do base quality recalibration.

4.4.1 Count Covariant

Currently, BisSNP only allow recalibration on 3 covariants: ReadGroupCovariate, QualityScoreCovariate and CycleCovariate:

java -Xmx4g -jar BisSNP-0.71.jar -R referenceGenome.fa -I sample.withRG.realigned.mdups.bam -T BisulfiteCountCovariates -knownSites dbsnp.vcf -cov ReadGroupCovariate -cov QualityScoreCovariate -cov CycleCovariate -recalFile recalFile_before.csv -nt cpu_cores_wanted

4.4.2 Write recalibrated base quality score into BAM file

Use the generated recalFile_before.csv for the recalibration and add recalibrated base quality score into BAM file:

java -Xmx4g -jar BisSNP-0.71.jar -R referenceGenome -I sample.withRG.realigned.mdups.bam
-o sample.withRG.realigned.mdups.recal.bam T BisulfiteTableRecalibration -recalFile recalFile_before.csv -maxQ 40

4.4.3 Re-Count Covariant

This step is to validate that the recalibration step is correct:

java -Xmx4g -jar BisSNP-0.71.jar -R referenceGenome.fa -I sample.withRG.realigned.mdups.recal.bam -T Bisulfite-CountCovariates -knownSites dbsnp.vcf -cov ReadGroupCovariate -cov QualityScoreCovariate -cov CycleCovariate -recalFile recalFile_after.csv -nt cpu_cores_wanted

4.4.4 Generate recalibration plot

Download BisulfiteAnalyzeCovariates.jar from our website: `http://epigenome.usc.edu/publicationdata/bissnp2011/utilies.html`.Then use the following command to generate plot that shows base quality score distribution before and after recalibration steps (R is required to be installed in your machine):

java -Xmx4g -jar BisulfiteAnalyzeCovariates.jar -recalFile recalFile_before.csv -outputDir dir_1 -ignoreQ 5 --max_quality_score 40

java -Xmx4g -jar BisulfiteAnalyzeCovariates.jar -recalFile recalFile_after.csv -outputDir dir_2 -ignoreQ 5 --max_quality_score 40

Here is an example plot that before and after base quality score recalibration:

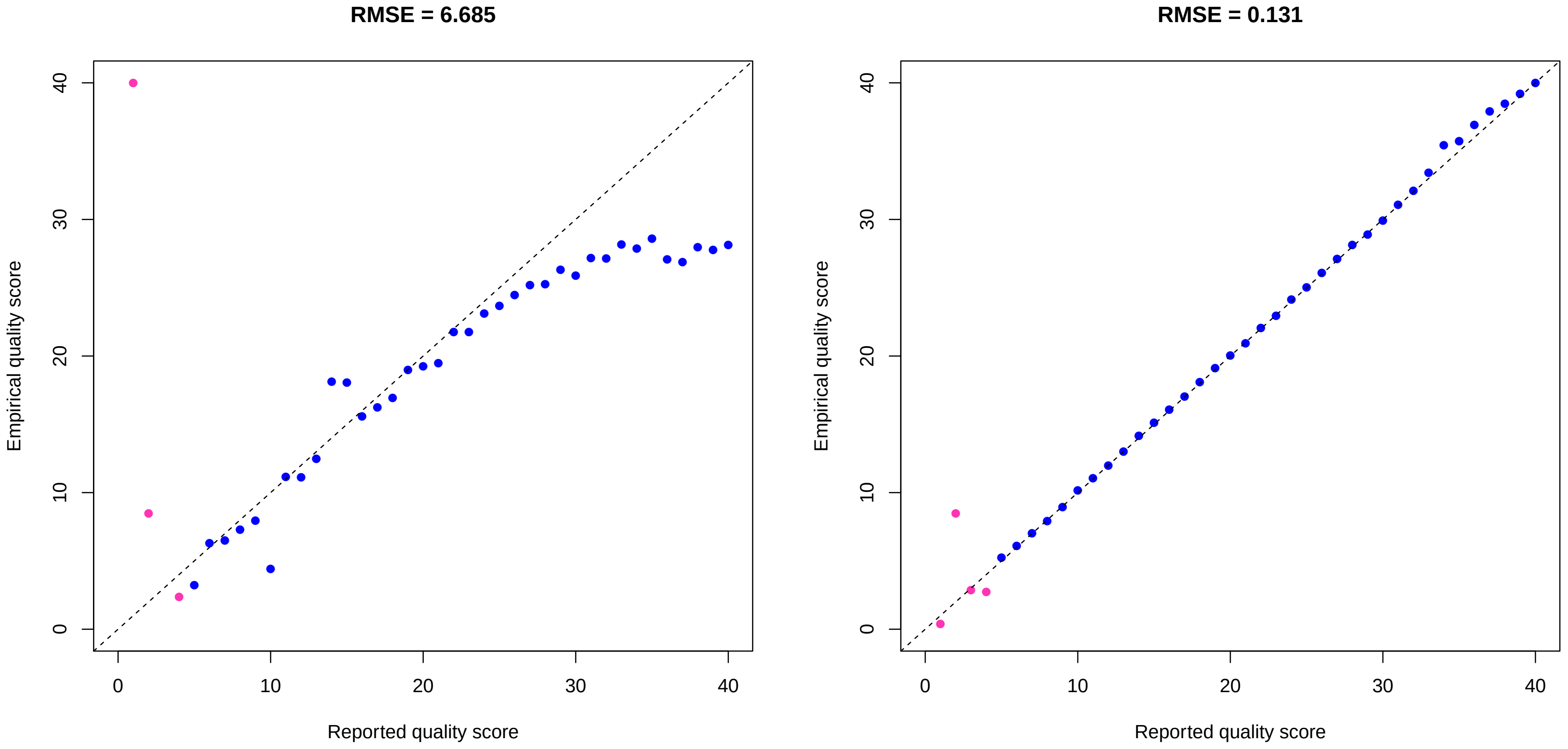

4.5 BisSNP genotyping

Here is the recommend criteria for genotyping by BisSNP:

java -Xmx4g -jar BisSNP-0.71.jar -R referenceGenome.fa -T BisulfiteGenotyper -I sample.withRG.realigned.mdups.recal.bam -D dbsnp.vcf -vfn1 cpg.raw.vcf -vfn2 snp.raw.vcf -L chr11:7000000-7100000 -stand_call_conf 20 -stand_emit_conf 0 -mmq 30 -mbq 0

After get the cpg.raw.vcf and snp.raw.vcf files, you should use the following script to sort vcf files by reference genome coordinates:

perl sortByRefAndCor.pl [--k chr_field_position] [--c coordinate_field_position] [--tmp dir] input_files referenceGenome.fa.fai

chr_field_position means the chromosome name's position in the input line (1-based), for .bed file, it is 1; coordinate_field_position means the coordinate's position in the input line (1-based), for .bed file, it is 2.

4.6 Filter fake SNPs

Because of Strutual variant, indel, copy number variation mapping bias or strand bias, there are still some of fake SNPs called. So we use the VCFpostprocessWalker commend to filter out some fake SNPs:

java -Xmx4g -jar BisSNP-0.71.jar -R referenceGenome.fa -T VCFpostprocess -oldVcf snp.raw.vcf -newVcf snp.filtered.vcf -snpVcf snp.raw.vcf -o snp.raw.filter.summary.txt

java -Xmx4g -jar BisSNP-0.71.jar -R referenceGenome.fa -T VCFpostprocess -oldVcf cpg.raw.vcf -newVcf cpg.filtered.vcf -snpVcf snp.raw.vcf -o cpg.raw.filter.summary.txt

VCFpostprocessWalker's options:

-qual or --genotype_qual
: genotype quality score filter for heterozygous SNP, default: 20

-sb or --strand_bias
: strand bias filter for heterozygous SNP, default: -0.02

-minCT or --min_ct_coverage
: minimum number of CT reads for count methylation level, default: 0

-maxCov or --max_coverage
: maximum coverage filter for heterozygous SNP, default: 120

-qd or --quality_by_depth
: quality by depth filter for heterozygous SNP, default: 1.0

-mq0 or --mapping_quality_zero

fraction of mapping_quality_zero filter for heterozygous SNP, default: 0.1

-minSNPinWind

minimum number of SNPs in the window, default:2

-windSizeForSNPfilter

window size for detect SNP cluster, default:10, means +/- 10bp distance, no second SNP there

-minBQ or --min_bq

minimum base quality for both of strand, default: 10, not enable this option yet

4.7 Generate bed file or wig file for SNP/DNA methylation visualization

You could specify any cytosine context instead of CG. e.g. extract GCH for NOMe-seq.

4.7.1 Converted to .wig format

perl vcf2wig.pl cpg.filtered.vcf CG

CG here could be any other context you want to extract, like GCH for NOMe-seq.

4.7.2 Converted to .bed format

perl vcf2bed.pl cpg.filtered.vcf CG

CG here could be any other context you want to extract, like GCH for NOMe-seq.

4.7.3 Converted to .bedgraph format

perl vcf2bedgraph.pl cpg.filtered.vcf CG

CG here could be any other context you want to extract, like GCH for NOMe-seq.

4.7.4 Extract cytosine coverage information to .bedgraph format

perl vcf2bedGraph.pl cpg.filtered.vcf CG

CG here could be any other context you want to extract, like GCH for NOMe-seq.

5 Interpret output files

Bis-SNP output TCGA VCF 1.1 file which is a subset of VCF4.1 format(for VCF4.1 refer to `http://www.1000genomes.org/wiki/Analysis/Variant%20Call%20Format/vcf-variant-call-format-version-41`, for TCGA

VCF 1.1 refer to `https://wiki.nci.nih.gov/display/TCGA/TCGA+Variant+Call+Format+(VCF)+1.1+Specification#TCGAVariantCallFormat%28VCF%291.1Specification-metainfo`). In the INFO and FORMAT column, there is some new format that are generated specifically in Bis-SNP:

5.1 VCF file

5.1.1 INFO column

Context: Cytosine pattern. like "CG" for homozygous CpG, "CR" for heterozygous CpG (CpA/CpG). The code rule obeys IUPAC code rule `http://www.bioinformatics.org/sms/iupac.html`. It is the summary across all of the samples.

CS: Cytosine pattern's strand.

5.1.2 FORMAT column

CP: The Best Cytosine pattern for this sample. like "CG" for homozygous CpG, "CR" for heterozygous CpG (CpA/CpG).

CM: Number of Cytosine reads(methylated) in this Cytosine position.

CU: Number of Thymine reads(unmethylated) in this Cytosine position.

BRC6: Bisulfite read counts: 1) number of C in cytosine strand, 2) number of T in cytosine strand, 3) number of A/G/N in cytosine strand, 4) number of G in guanine strand, 5) number of A in guanine strand, 6) number of C/T/N in guanine strand

5.2 CpG reads file

Output each CpG in each reads. The CpG in the same reads would have the same readID. Here is the explanation of each column:

chr: chromosome name

pos: genomic coordinate.(1-based)

methyStatus: methylation status of this CpG. m: methylated. u: unmethylated

baseQ: phredscale base quality of the Cytosine in this CpG site.

strand: strand of this CpG.

readID: encrypt readID. The same reads would have the same readID

6 Additional useful script

Some additional useful perl scripts to convert from VCF file to methylation CpG bed file or wiggle tract file for visualization. The detailed information refer to `http://epigenome.usc.edu/publicationdata/bissnp2011/utilies.html`

7 BisSNP API structure

This part is for other programmer to build tools on top of BisSNP genotype engine. Java API doc will be available soon.

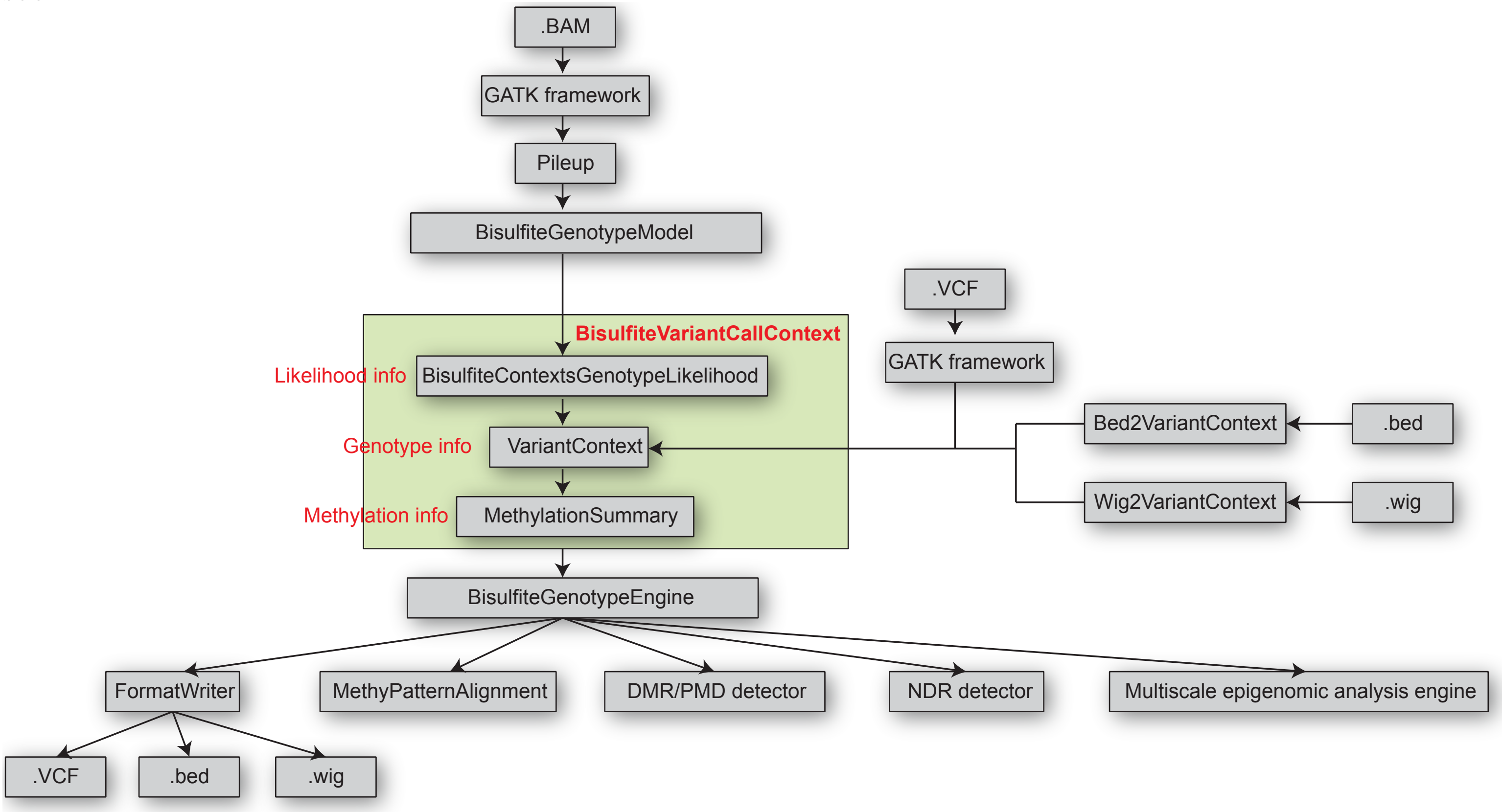

8 Build on source code

Source code is available on SourceForge website, which could be checkout by command:
"svn checkout svn://svn.code.sf.net/p/bissnp/code/trunk"
All of the required libraries are available in `http://svn.code.sf.net/p/bissnp/code/trunk/lib/`.

9 Contact for help

For any of question on Bis-SNP, please send email to lyping1986@gmail.com or benbfly@gamil.com
